# Radon indoors source potential from soil gas in a temperate climate: impact of infiltration rate and seismicity

**DOI:** 10.1007/s11356-024-32334-w

**Published:** 2024-02-19

**Authors:** Stylianos Stoulos

**Affiliations:** Nuclear Physics Lab, School of Physics, Aristotle University of Thessaloniki, 54124 Thessaloniki, Greece

**Keywords:** Radon entry indoors, Soil, Infiltration rate, Seismicity, Water, Radon source potential

## Abstract

Indoor radon source potential from unground soil was monitored using prototype devices approaching a dwelling with a cellar basement at 1 depth from the soil-atmosphere interface. Therefore, the radon concentrations in soil gas were monitored at 1 m depth. Integrated radon measurements were performed, and the results correlated with meteorological parameters. The influence of the difference in outdoor and device-soil temperature was considered, and the infiltration rate was calculated. The effect of the soil temperature gradient on the soil radon entry rate was evaluated. The indoor radon entry rate due to the soil gas was 7.0 ± 2.7 Bq m^−3^ h^−1^. The radon entry rate was 5.0 ± 0.8 Bq m^−3^ h^−1^ due to diffusion. In contrast, the advection-drive flow of soil gas is ranged up to ± 4.0 Bq m^−3^ h^−1^. So, the infiltration rate of the model dwelling was 0.7 (± 0.5) × 10^−1^ h^−1^ if only the stack effect occurred. The radon levels in tap water were measured, and the radon entry rate was estimated at 1.3 ± 0.7 Bq m^−3^ h^−1^. If the ventilation rate is low or seismic faulting appears, the soil radon entry is increased by one order of magnitude. The soil radon appeared like the building materials, having 1/3 of the total indoor radon entry, while outdoor air was slightly lower (28%), with tap water at 5%. The resident’s mortality risk occurred at < 2.5% for typical dwellings in temperate climate areas founded on sand-gravel underground. The risk rises to 34% with an extremely low ventilation rate between indoors and outdoors or high radon entry from the soil due to seismic faulting.

## Introduction

Extensive knowledge on indoor radon problems has been accumulated during the last decades since about half of the annual absorbed dose in a normal environment is due to radon and its decay products deposited in the human respiratory system. Epidemiological studies demonstrate that the resident’s risk of lung cancer increased by 5–12% per 100 Bq m^−3^ enhancement on measured indoor radon concentration following a linear relation even when the radon level was lower than 200 Bq m^−3^ (Darby et al. 2005; Krewski et al. 2005; UNSCEAR 2006; 2017; 2019). That indoor radon concentration is the average value that has been recommended as an action level by the ICRP, WHO and EU for future dwellings, while for the existing dwellings, the respective level ranged from two up to four times higher [WHO 2009; ICRP 2017; UNSCEAR 2020/21]. The adopted limit on the annual internal effective dose of 1.6 mSv due to radon progenies attached to the lung and received by the public keeps the resident’s risk of lung cancer lower than 5%.

Ground soil is the primary source of indoor radon, except when building materials seriously loaded with natural radioactivity are used (Nero and Nazaroff 1984). So, many natural radioactivity and indoor radon measurements and models have been performed and proposed for the last 50 years studying radon transport indoors, mainly from the soil and building materials, but also from atmospheric air, tap water and gas systems, contributing to the least. All this research has been discussed and evaluated by national and international authorities who gave suggestions after risk assessment (UNSCEAR 2000; 20/21; WHO 2009; ICRP 2017). The ground soil type, masonry construction and age influence indoor radon entry because they define the porosity and thermal conductivity that affect the permeability of the dwelling and the infiltration rate (Mowris and Fisk 1988).

Expect these radon sources and the inhabitants to play a key role in radon indoor levels. Their habits and lifestyle define the risk they take from radon daughters’ inhalation. The residents’ usage frequency of tap water and gas identifies the potential radon entry indoors. Opening windows and doors is the most influencing habit since the inserted low-radon outdoor air dilutes the high radon level accumulated indoors. This effect of the natural ventilation rate is eliminated when air conditioning systems are used (Mowris and Fisk 1988; UNSCEAR 2000; 2020/21). Seasonal and daily variations of these rates are detected based on typical meteorological parameter variation, such as rainfall, wind speed, outdoor and indoor temperature and the soil and wall temperature gradient (Robinson and Sextro 1995; Nazaroff 1992; Arvela et al. 2015). Managing so many parameters is why indoor radon remains an interesting subject.

The approaches to indoor radon entry due to soil gas in a commonly adopted model dwelling must consider data on the radon level inside the soil porosity and radon effective diffusion coefficient (Robinson and Sextro 1995; UNSCEAR 2000; Font and Baixeras 2003). The water in soil porosity dramatically decreases the radon transport in soil because water has three orders of a magnitude lower diffusion coefficient than air. Besides, indoor soil radon entry is strongly influenced by the soil temperature gradient between the soil-air interface in the cellar basement and the upper and lower ground levels (Mowris and Fisk 1988; Nazaroff 1992). The indoor-to-outdoor temperature difference impacts the final amount of radon that remains indoors. Because the above difference depends on the residents’ habits of using natural ventilation, the approach should be implemented as the dwelling is uninhabited. Therefore, the infiltration-ventilation rate of the dwelling can be estimated by giving information on the indoor air quality count of risky gases coming underground (Nero and Nazaroff 1984).

The present study follows the above approach, measuring radon concentrations in soil gas and radon entry rate in a prototype device approaching a model dwelling with a cellar basement at 1 m depth. The soil grains’ natural radioactivity and radon emanation factor have been measured using γ-ray spectrometry. Integrated measurements using solid-state nuclear track detectors (SSNTD) were performed, and the results were correlated with meteorological parameters. The effect of the soil temperature gradient and the influence of the stack effect due to the difference in outdoor and device-soil temperature on the soil radon entry rate was discussed. Likewise, the infiltration rate was calculated in a typical dwelling based on the common sand-gravel ground in the temperate climate of the Mediterranean city of Thessaloniki, Greece. Besides, the area’s seismic impact on soil radon entry indoors was evaluated based on measurements along active faults. Moreover, the radon concentration in tap water was determined using Luca’s scintillation measurements daily and monthly, and the radon entry rate was estimated. Accomplishing the above radon entry rates with the entry due to building materials and atmospheric air, from previous studies, the radon source potential indoors of the model dwelling was discussed.

## Instrumentation and methods

The measurements were performed on the university campus of AUTH, in an open space where the meteorological parameter monitoring takes place by the Observatory of Geological School. The same meteorological data were used in the manuscript analysis. The tap water was collected in the laboratory from the city’s public water system.

The general transport equation of radon-soil fluid through the underground soil due to diffusion and advection process is (Nazarrof 1992; Antonopoulos-Ntomis et al. 2009):

1 $$\frac{\partial C}{\partial t}=D\frac{{\partial }^{2}C}{{\partial }^{2}x}-v\frac{\partial C}{\partial x}-\lambda C+R$$

where *C* is the radon activity concentration in the gas phase (Bq m^−3^ of pore air), *D* is the effective diffusion coefficient (m^2^ s^−1^), *v* is the effective advective velocity of radon in soil pore and cracks (m s^−1^) and *λ* is the radon decay constant (s^−1^). The radon emanation rate or radon entry rate in the porous system (*R*, Bq m^−3^ s^−1^) is equal to the *E* (emanation factor) × *λ*_Ra_ (radium decay constant) × *A*_Ra_ (radium activity of soil, Bq kg^−1^) × *ρ* (soil density, kg m^−3^). If only an advection appears, then the advection length is *L*_A_ = *u* / *λ* and can range up to kilometers. If only diffusion occurred, the radon diffusion length is given *L*_D_ = √ (*D* / *λ*) and in soil ranged from 7 to 110 cm depending on the soil type. If both of them are taken into account, then an effective coefficient can be assumed, and the radon effective length is *L*_D_ = √ (*D*_eff_ / λ) = 1 / (√ [(*u* / 2*D*)^2^ + (*λ* / *D*)] − (*u* / 2*D*).

The ground soil in the studied location consisted mostly of psammites and gravels with a radon diffusion length of 70–110 cm (Table 1). In contrast, materials like PVC, plexiglass and epoxy glue are less porous, with diffusion lengths of 0.22–0.07 cm (UNSCEAR 2000). Therefore, these materials are chosen for this study’s specially made radon devices to reduce leakage. Moreover, each time the top of the device opened to replace the SSNTD, it closed using silicone and plastic tape.

**Table 1:** The radon diffusion coefficient and length for materials involved in this study

Material	Diffusion coef. (*D*, cm^2^ s^−1^)	Diffusion length (*L*_D_, cm)
Air	1 × 10^−1^	220
Water	1 × 10^−5^	2.2
Sand	1 × 10^−3^ − 1 × 10^−2^	22 − 70
Sand + gravel	5 × 10^−2^	110
Silty sand	1 × 10^−2^	70
Silt − concrete aged	1 × 10^−3^	22
Clay − concrete	1 × 10^−4^	7
Asphalt − asbestos	1 × 10^−6^	0.7
PVC − Plexiglass − silicone	1 × 10^−7^ − 1 × 10^−8^	0.22 − 0.07
Epoxy	1 × 10^−8^	0.07

**Fig. 1:**
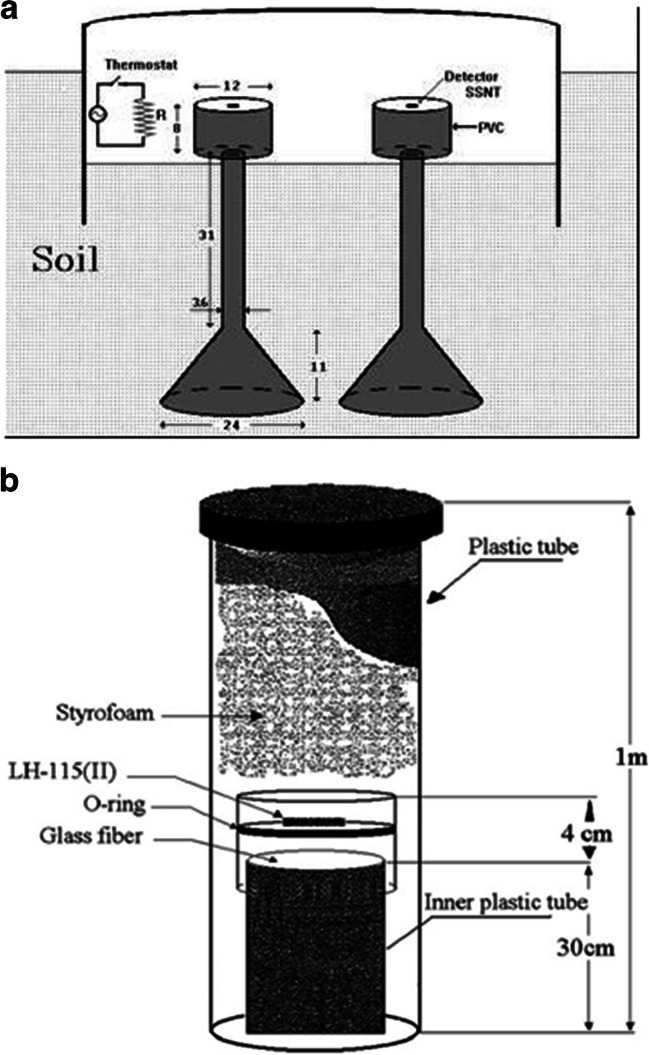
The prototype device used for (**a**) radon entry rate measurements from the soil and (**b)** radon concentration measurements in the soil

The indoor soil radon entry was estimated using the radon exhalation rate measured from a 1-m underground soil surface. Exhalation rate measurements were performed with a specially designed device (Fig. 1a), where radon penetrated the device from its open base, placed underground and measured at its upper part on the soil-air interface (Savvides et al. 1985). The detector was at an appropriate height from the underground surface, so the thoron could not reach it. A thermostat was used in the upper part of the device to keep thermal conditions comparable with those in the cellar basement of a dwelling. Likewise, it also reduces any influence from moisture accumulation on the detector surface. The measured exhalation rate transformed the device’s radon entry rate by applying the surface-to-volume ratio of the cellar basement.

Assuming that *N*_0_ is the radon nuclei per cubic meter in the soil, and N the nuclei per cubic meter in the radon device–basement, then assuming that the radon outdoor contribution is negligible, the variation of Rn nuclei in the device is:

2 $${~}^{dN}\!\left/ \!{~}_{dt}\right.= {\lambda }_{{\mathrm{leak}}-{\mathrm{vent}}} {N}_{{\mathrm{o}}}- \lambda C-{\lambda }_{{\mathrm{leak}}-{\mathrm{vent}}} N$$

3 $$\lambda N= {\lambda }_{{\mathrm{leak}}-{\mathrm{vent}}} {N}_{{\mathrm{o}}} (1- {e}^{- \left(\uplambda +{\uplambda }_{{\mathrm{leak}}-{\mathrm{vent}}}\right)t})$$

4 $$C= \frac{{\lambda }_{{\mathrm{leak}}-{\mathrm{vent}}}}{\lambda } {C}_{{\mathrm{o}}} (1- {e}^{- \left(\uplambda +{\uplambda }_{{\mathrm{leak}}-{\mathrm{vent}}}\right)t})$$

where *λ*_leak-vent_ is the Rn leakage due to device ventilation and λ the radon decay constant. The Rn leakage rate can be calculated if the radon concentration in soil gas is known. Therefore, radon in soil gas was also monitored for 15 days by LR-115 Kodak SSNTD (integrated radon measurements) in specially made devices consisting of a plastic tube, 44 mm inner diameter, 50 mm outer diam., and 300 mm in length, with the detectors being on top of the tube (Fig. 1b). The plastic tube with the radon detector was set inside another plastic tube of 1000 mm in length with a 70 mm inner diameter. The space between the two plastic tubes was filled with granular Styrofoam material to thermally isolate the devices. To avoid the thoron and radon daughters’ entry into the measuring chamber, a glass fibre–type GF/B was used.

The radon concentration was determined using the track density measured and the device’s efficiency. After irradiation, the SSNTD was etched using a 6.25N NaOH solution at 60 °C for 2 h. The track density was measured using an optical microscope. The devices were efficiency-calibrated using a radon chamber of 110 l with a leakage rate two orders of magnitude lower than the radon decay constant and an Rn source of 20 kBq (Stoulos et al. 2003). The weighted calibration uncertainty was 11% after five irradiations, and the track counting ranged from 14 to 19%.

The radon concentration in the public water system was measured by a proper vacuum water degassing system (WG-1001, PYLON). The radon concentration in water samples was determined with a scintillation flask—Lucas cell. Radon was measured by alpha counting using appropriate Luca’s scintillation cells. The spectrometer was linked to a portable radon monitor, AB-5 (PYLON). This trace environmental level radon gas detector detects radon levels as low as 11 Bq m^−3^. The volume of Lucas cells is 270 ml with ZnS (Ag), an active area of 27 700 mm^2^, a counting efficiency of 0.75 ± 0.02, and a sensitivity of 0.037 cpm Bq^−1^m^3^. The measurement uncertainty was 9–10%, mostly due to calibration error; the counting error was 1–2%. No radon concentration measurements in the gas supply system were performed since the gas burner is located outside the Greek dwellings, so no influence on indoor radon level is expected.

The natural radioactivity levels and radon emanation factor from soil grains were determined using high-resolution gamma-ray spectrometry systems. The samples were measured using a high-resolution gamma-ray spectrometry system consisting of a high purity Ge (HPGe) coaxial detector with 20% efficiency and 1.8 keV resolutions at 1.33 MeV photons, shielded by 10.2 cm of Pb, 1 mm Cd and 1 mm Cu. The determination of ^226^Ra content was based on measuring the radon decay products being in equilibrium. The accuracy depends on the integral trapping of radon gas in the sample volume. So, 2% w of charcoal powder (smaller than 400 µm) was mixed with the sample before its hermetic sealing and storage for 30 days in a freezer. The mean value of ^214^Pb concentration was estimated using 295 and 352 keV γ-rays and ^214^Bi, using 609, 1120 and 1764 keV γ-rays.

The radon emanation factor, defined as radon escapes to production ratio, was determined by measuring (i) the ^226^Ra concentration after mixing charcoal with the sample, sealing it hermetically and storing it in a freezer during the radon in-growth period and (ii) the concentration in the sample measured placing the sample in an open vessel with no charcoal addition (Stoulos et al. 2004). The efficiency calibration of the spectrometry systems was performed with the radionuclide-specific efficiency method. A set of high-quality certified reference materials (RGU-1) was used to reduce the uncertainty and the influence of coincidence summation and self-absorption effects of the emitted gamma photons in the sample (Gilmore and Hemingway 1995). The measuring time was 200,000 s, and the MDA of ^226^Ra was 2 Bq kg^−1^. The uncertainty of counting statistics was 2–5%, like the efficiency calibration.

## Results and discussion

**Fig. 2:**
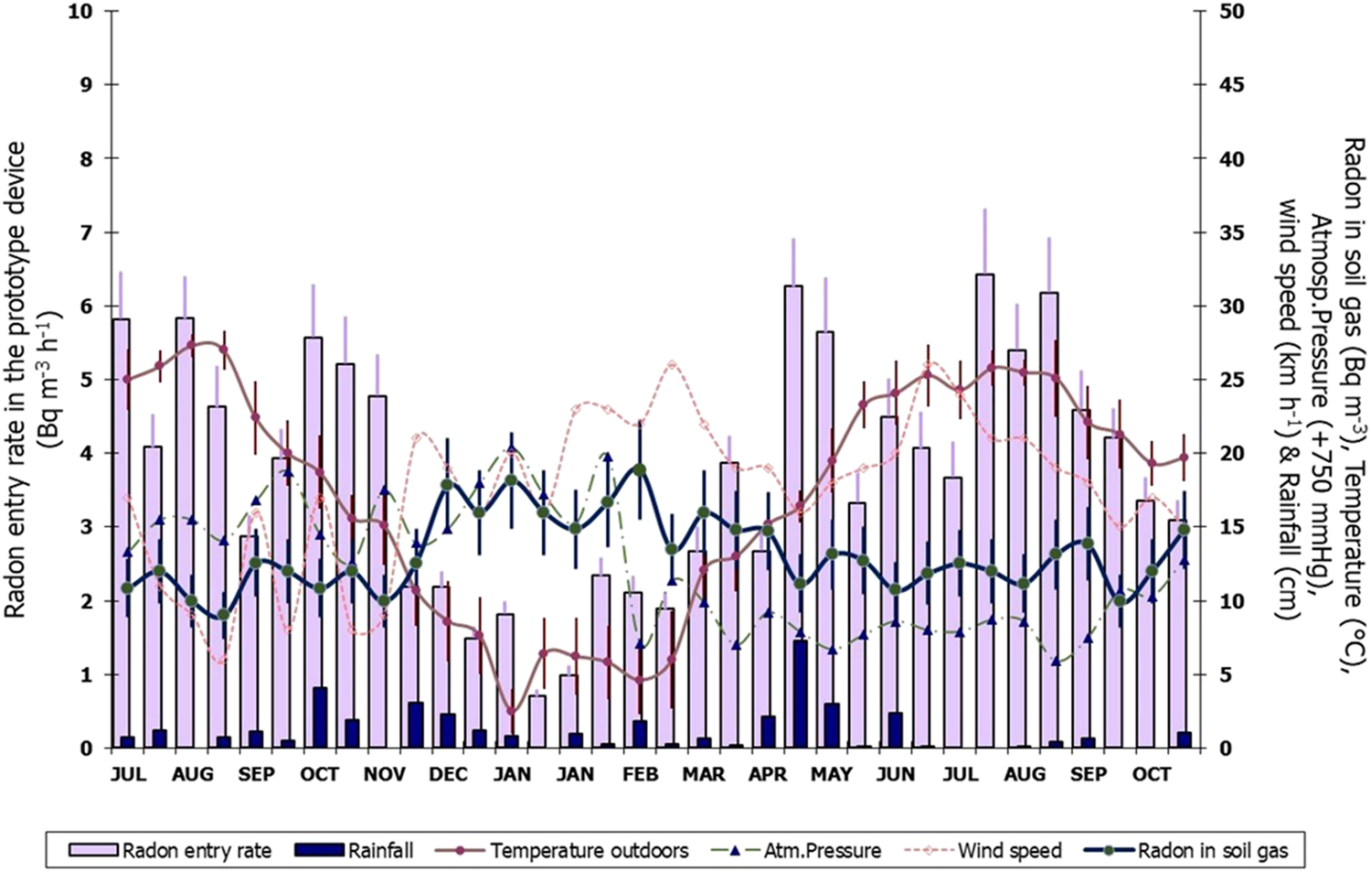
The radon entry rate and concentration in soil gas measured in association with air temperature, pressure, wind speed and rainfall

The radon entry rate (Bq m^−3^ h^−1^) is estimated based on the radon exhalation rate measurements (Bq m^−2^ h^−1^) from a 1-m surface underground (ISO, 2016) multiplied by the surface-to-volume ratio 0.13 of the device. The radon entry data were correlated with meteorological parameters. Also, the radon concentration in the soil gas measured at a 1-m level in the ground was correlated with the same parameters. The data is given in Table S6 and shown in Fig. 2. The radon levels in underground soil are among the lowest values presented in the literature (UNSCEAR 2000; 2019).

**Fig. 3:**
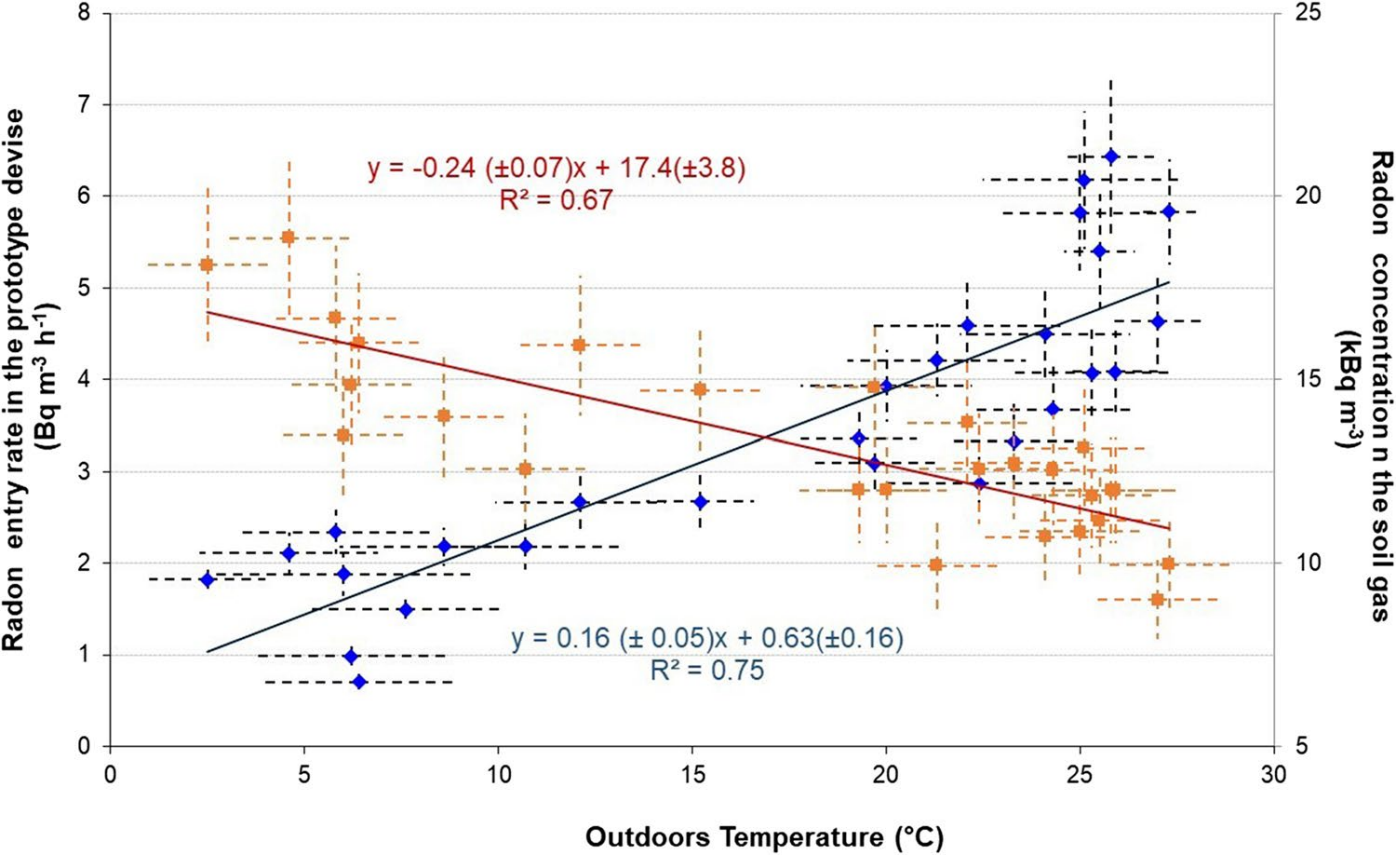
Radon soil entry rate (in blue points and line) and soil gas concentration correlation (in orange points and line) with the outdoor temperature

A significant correlation appears with the air temperature and no correlation with wind speed and atmospheric pressure (Fig. 3). The highest soil radon entry rate appeared in the summertime, 6 Bq m^−3^ h^−1^, and the lowest, 1 Bq m^−3^ h^−1^, in wintertime. According to the relationship between the radon entry and the outdoor temperature obtained by *R*^2^ = 75%, when the outdoor temperature is 0 °C, the radon entry is 0.63 ± 0.18 Bq m^−3^ h^−1^. An increase of 1 °C in outdoor temperature produces radon entry rates of 0.16 ± 0.05 Bq m^−3^ h^−1^. This correlation is due to the stack effect of temperature differences outdoors and in the device. This effect produces an infiltration rate (h^−1^) between the ground soil gas entering the device from 1 m underground (cellar basement) and the outdoor atmosphere. The infiltration rate is similar to the radon leakage or ventilation rate in Eq. 4. The correlation is strongly impacted when heavy rainfall occurs with heights of centimeter (Arvela et al. 2015). Then, a higher radon entry rate than that was detected if only outdoor temperature influence is considered. Water inserts in the soil porosity and increases the barometric pressure inside the soil porosity around the device. So, radon is driven through the device since no barometric pressure is presented below, unaffected by the rainwater, giving the highest value measured 5 − 6 Bq m^−3^ h^−1^.

On the contrary, in soil gas, the radon concentration (kBq m^−3^) is inversely correlated with outdoor temperature (Fig. 3), like that reported by King and Minissale (1994). In the relationship obtained by *R*^2^ = 67%, once the temperature is 0 °C, the radon in soil gas is 17.4 ± 3.8 kBq m^−3^. An increase of 1 °C in outdoor temperature decreases radon concentration in soil gas by − 0.24 ± 0.07 kBq m^−3^. The average value of radon activity concentration in soil gas is estimated as 13.2 ± 6.2 kBq m^−3^. During summertime, the fresh, warm atmospheric air penetrating the porous soil dilutes the cold radon in soil gas, giving lower radon levels (7–14 kBq m^−3^). In wintertime, high radon levels are inside the ground (13–19 kBq m^−3^) when the warm soil gas exhales into the cold atmosphere (Fleischer and Mogro-Campero 1978; King 1980; Nazaroff 1992). Barometric pressure also affects radon transport in the soil. When the radon pressure-driven flow (advection) dominates over diffusion, the soil gas can move long distances (Clements and Wilkening 1974; Shapiro et al. 1985; Nazaroff 1992). A lower atmospheric pressure due to a very strong wind makes radon soil gas enter the atmosphere through this pumping effect, and the radon level in soil gas is significantly reduced. However, the data on radon concentrations in soil gas obtained in this study does not correlate with wind speed or atmospheric pressure.

### Radon entry due to infiltration rate

**Fig. 4:**
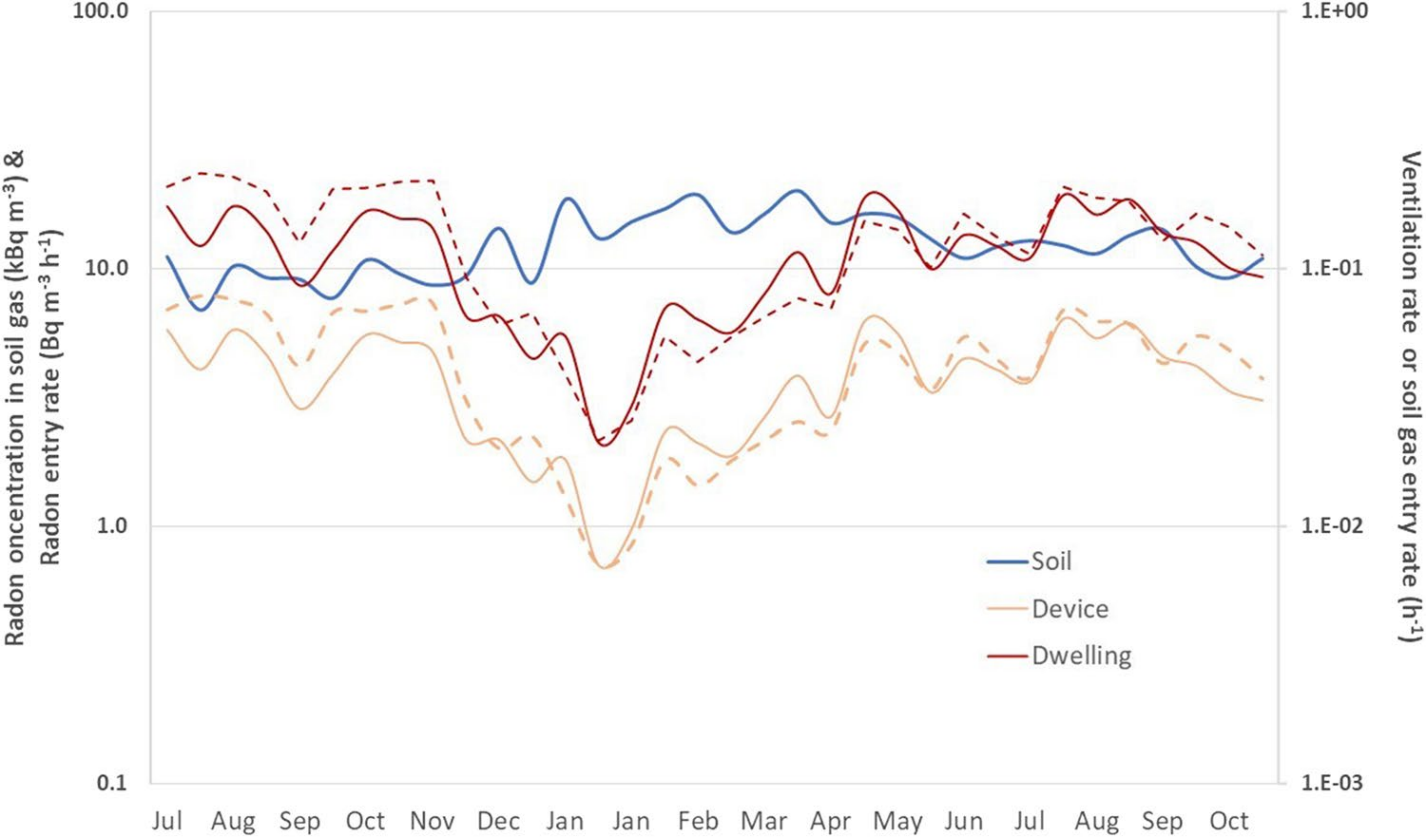
The infiltration rate estimate for the prototype device and the model dwelling in dash lines. The radon soil gas level and radon entry rate measured are in solid lines

The infiltration rate, h^−1^, between porous soil and the device–cellar basement is estimated by Eq. 4, with the exponential component since the exposure time was 3 times the mean life of radon. The results are shown in Table S7 and Fig. 4, along with the radon concentration data in soil gas. The highest infiltration rate, 7.9 × 10^−2^ h^−1^, appeared in the summertime, and the lowest, 0.7 × 10^−2^ h^−1^, in the wintertime. The mean annual infiltration rate was 3.8 (± 2.6) × 10^−2^ h^−1^. The infiltration rate strongly depends on the surface-to-volume (S/V) ratio since the estimated radon entry rate comes from multiplying the measured radon exhalation rate from the ground with the S/V of the device. The device has a S/V ratio of 0.13. Suppose a prototype dwelling (Table 2) with only the outer masonry and a cellar basement of 1 m with a living chamber of 2.8 m in height. In such a case, the S/V ratio is 0.26. The mean annual infiltration rate is now 0.7 (± 0.5) × 10^−1^ h^−1^ due to the stack effect occurring via indoor–outdoor temperature difference (Fig. 7) because the radon entry rate from the ground indoors became higher, ranging from 1.1 to 8.6 Bq m^−3^ h^−1^ while the soil gas radon concentration remains the same. The radon infiltration rates are among the typical values in the literature for an inhabitant dwelling with an extremely low ventilation rate (Nazaroff 1992; Robinson and Sextro 1995; UNSCEAR 2000).

**Table 2:** Parameters of the adopted model residential dwelling

Volume	10 × 10 × 2.8 m^3^
Rooms arrangement	2 rooms [4 × 4 × 2.8 m^3^] 1 room [2 × 2 × 2.8 m^3^]
Concrete blocks	Floor, ceiling and 15% of the walls
Clay bricks	85% of the walls
Windows–doors	10% of the room surface
Wall thickness Floor/ceiling thickness	0.2 m 0.25 m
Foundation depth	1 m
Air exchange rate (*λ*_v_)	1 h^−1^ (mean annual value)
Number of residents	4

The seasonal variation of infiltration rate is the same as the radon soil entry rate and is inversely correlated with the radon level in soil gas. The indoor–outdoor temperature difference transports radon soil gas throughout the device-cellar basement to the atmosphere and vice versa. In wintertime, the higher the indoor temperature, the lower the radon entry from the cold ground soil due to a lower infiltration rate (Mowris and Fisk 1988; Nazaroff 1992). The radon entry rate measured corresponds to a non-resident dwelling with a cellar basement at a 1 m depth. The habits and lifestyle of the residents play a key role in the radon centration indoors. How often are the windows–doors open so fresh low radon atmospheric air comes inside? And if they are using air-conditioning, especially in summer, it affects the ventilation rate of the dwelling. The infiltration rate is lower than the ventilation rate. The annual average ventilation rate of 1.0 ± 0.5 h^−1^ is adopted (UNSCEAR 2000; 2020/21).

The winter-to-summer ratio (W/S) of indoor radon concentrations ranges from 1.4 to 2.0 (UNSCEAR 2000; WHO 2009), which is anti-correlated with the radon entry rate. Natural ventilation eliminates the higher soil radon gas entries in an uninhabited dwelling during the summer due to residents’ habits of opening doors and windows. In contrast, in wintertime, the heating of the dwelling and the closed windows and doors accumulate low radon entry from the soil, giving high indoor radon concentrations. The cooling residential ventilation due to air conditions also almost eliminates the manual ventilation rate to reduce thermal loss. During the summer, the highest radon soil entry rate and the highest positive Δ*T* soil indoors lead to the highest radon entry from the soil. Therefore, combining the affected rates give radon indoor concentrations higher than in winter.

**Fig. 5:**
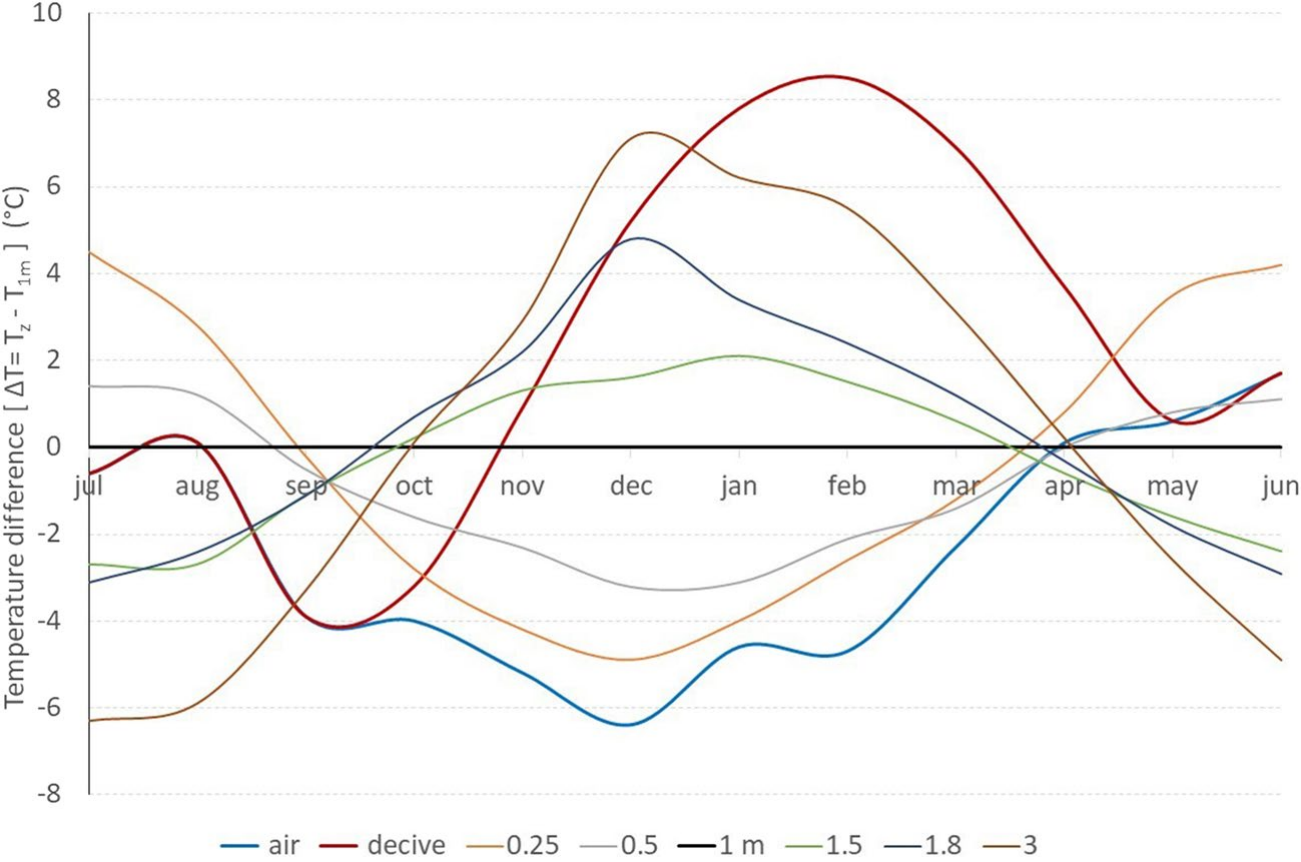
The soil temperature gradient and the device-dwelling and air

The thermostat in the upper part of the radon entry device (Fig. 1) regulated the temperature at 18 °C from October to April, as usually applied in the cellar basement of a residential dwelling. During the rest period, the thermostat was closed, and the temperature inside the device was the same as the outdoor air (see Table S8). The table also presented the soil temperature variations and gradient in the ground from the surface to 3 m and the monthly average radon entry from the ground as estimated using the prototype dwelling. The data is now the monthly average soil temperature given by the Observatory measurements and the corresponding monthly average of radon entry rate. The zero-reference surface was − 1 m, from which radon enters the cellar basement. The soil temperature gradient per meter of ground soil is presented in Fig. 5. The figure also illustrates the temperature difference between the 1-m surface, the outdoor and the device temperature. The influence of inhabitant habits on heating the cellar basement during wintertime is evident (Robinson and Sextro 1995).

**Fig. 6:**
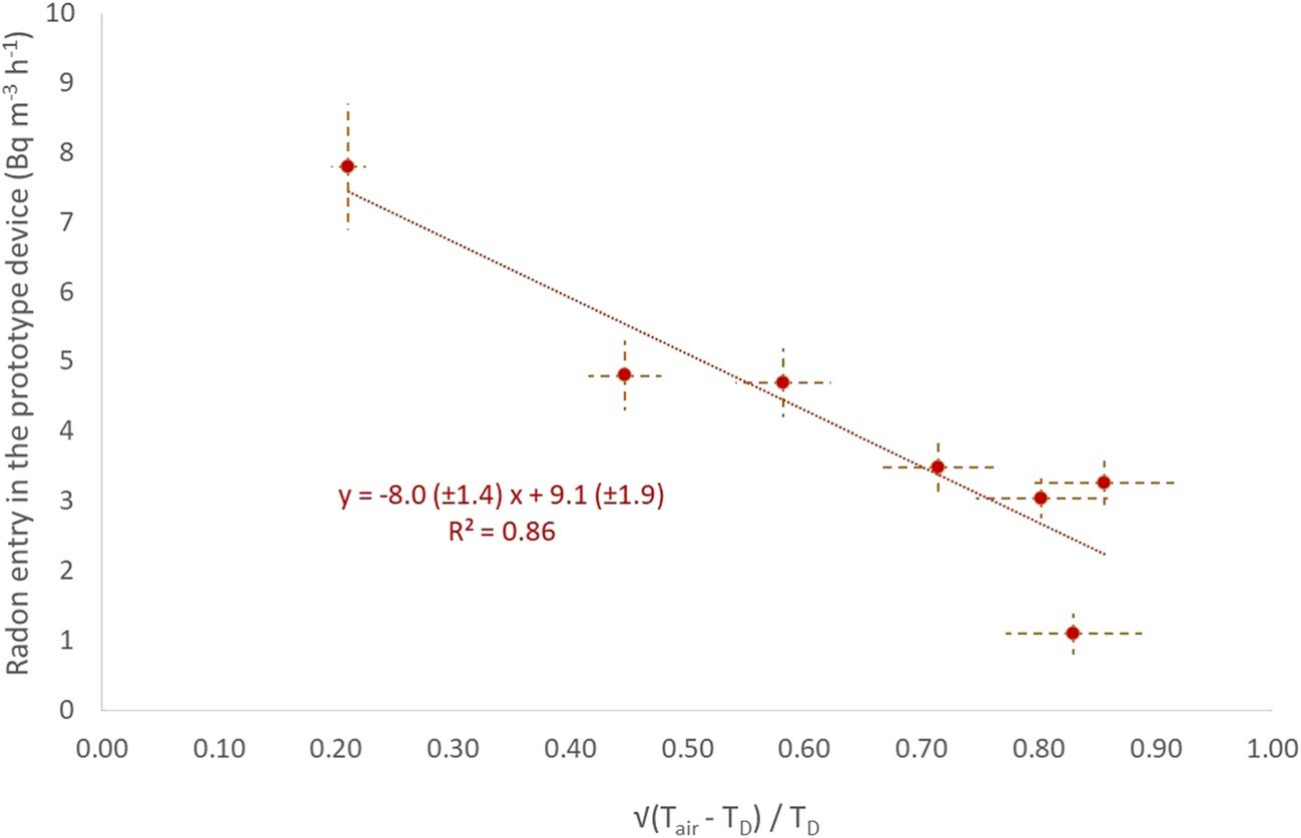
The impact of stack effect on indoor radon entry from the underground soil

The following evaluation did not consider the radon entry rate in months with high rainfall because it strongly affected the radon entry rate from soil indoors. From October to April, the stack effect occurred due to heating application since there is Δ*T* = *T*_out_ − *T*_0_. The temperature *T*_0_ is the temperature at 1 m depth of soil. The radon soil entry rate correlated in Fig. 6 to the square root of the Δ*T*/*T*_0_ since the stack effect is an analogue to this parameter (Mowris and Fisk 1988). A strong inverse correlation (*R*^2^ = 86%) was presented, and the radon entry rate was 9.1 ± 1.9 Bq m^−3^ h^−1^ when no temperature difference indoors–outdoors appeared, like those values measured during summertime. The radon entry rate slope is estimated as − 8.0 ± 1.4 Bq m^−3^ h^−1^ per Δ*T*/*T*_0_. When (*T*_out_ − *T*_0_) equals *T*_0_, the lowest radon entry rate was 1.1 ± 2.8 Bq m^−3^ h^−1^, as measured in January (see Table S8).

**Fig. 7:**
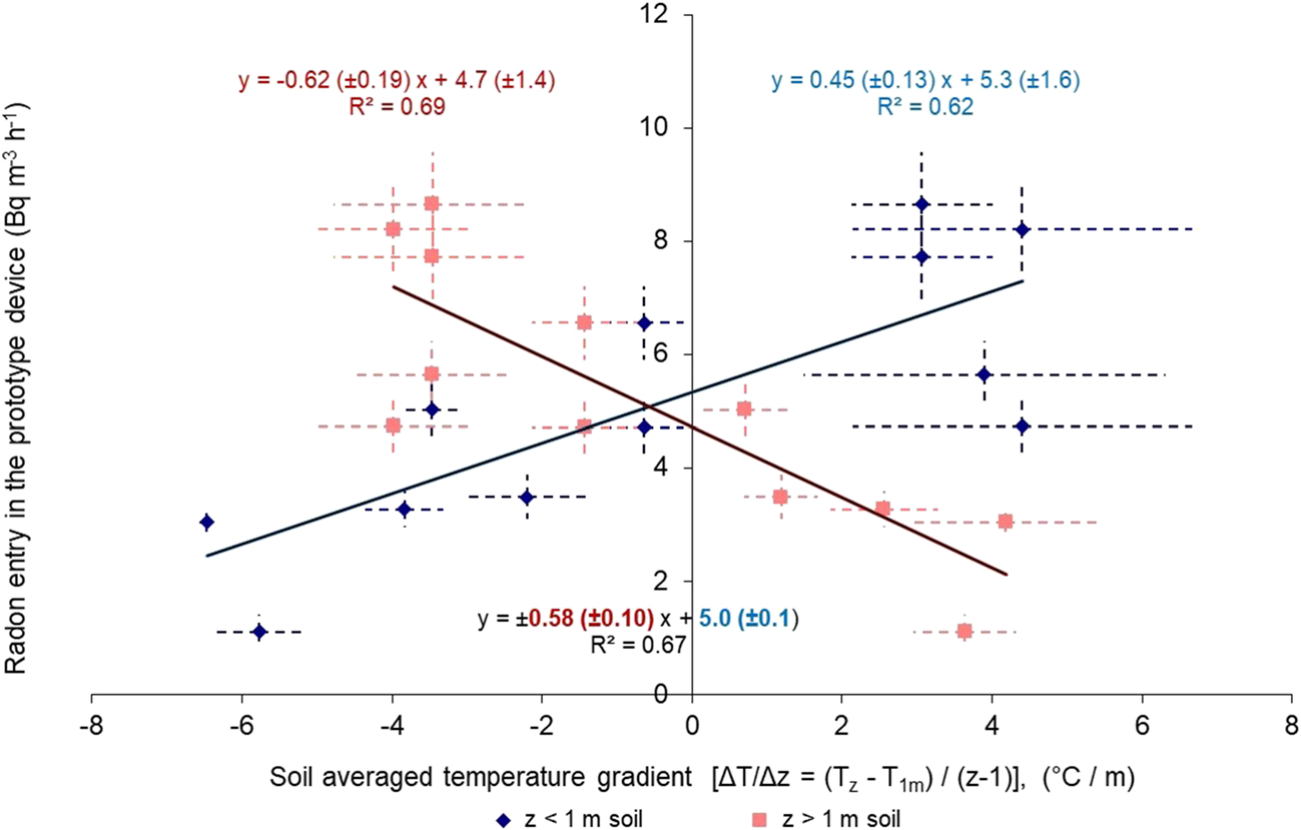
The radon entry rate correlation to the soil temperature gradient. The blue points and line are referred to the above 1-m soil layers (0–1 m), while the orange-dark to the below 1-m soil layers (1–3 m)

The soil temperature gradient also influences the radon entry rate into the cellar basement (Robinson and Sextro 1995), as shown in Fig. 7. A good inverse correlation (*R*^2^ = 67%) appeared for both up- and underlying soil of the basement 1 m above the surface. The expected inverse relationship between the upper and down layers concerning the soil gas flow has appeared. The influence of the under layers was stronger than the upper soil layers. When no soil temperature gradient appeared, the radon entry rate corresponded only to diffusion (Nazaroff 1992). This value was 5.0 ± 0.8 Bq m^−3^ h^−1^ as the annual mean value of radon entry rate from the ground indoors. Similar values were measured in April and October when the temperature was stable from 3 m deep in the ground to the basement and the atmosphere.

The slope was 0.58 ± 0.10 Bq m^−3^ h^−1^ per °C m^−1^, indicating the increase–decrease of the diffusion value due to the stack effect and the temperature-driven flow of soil gas into the basement. The slope can be expressed as the excess or loss in surface exhalation rate (Bq m^−2^ h^−1^) from the cellar-soil interface at 1 m depth, produced by each 1 °C difference between the soil and the basement. As the gradient was positive, radon entry was performed from the upper soil part, and the low contribution was from the underlying ground. The inverse pattern occurred when a negative soil temperature gradient appeared (Mowris and Fisk 1988; Nazaroff 1992; Robinson and Sextro 1995). Combining the above soil air flows, the radon soil entry rate in a prototype dwelling gives a minimum of 1.1 and a max of 8.6 Bq m^−3^ h^−1^ radon entry indoors of the prototype dwelling. So, the temperature-driven flow of soil gas into the basement contributed ± 3 Bq m^−3^ h^−1^.

The advection to diffusion ratio was ± 0.6 and was mostly affected by the climate conditions. For colder wintertime combined with hotter summertime, the driven flow of soil gas into the basement increases and surpasses diffusion given a ratio value > 1 and a double at the least radon entry indoors. Since the cooling system was applied, the advection-driven flow of soil gas from the warm soil to the cool dwelling amplifies the radon soil entry to high levels (Mowris and Fisk 1988). Another reason, except for the extreme climate conditions, is the area’s seismicity and uranium content underground, significantly impacting the radon entry rate indoors from the ground (UNSCEAR 2000). The high Greek seismicity gives high risks for radon problems indoors in many regions. In contrast, only two Greek regions have high natural radioactivity due to the high uranium content in Northern Greece near Lake Prespes and River Nestos.

### Impact of seismicity

The radon entry rate was measured at seismic areas in Thessaly, Central Greece, where severe earthquakes of 6.5 M occurred in 2021 and another 7 M in 1954 (Seismological Station AUTH, 2023). Three radon stations using the radon device presented in Fig. 1a were placed in an open space in Ekkara, and the other was near the public water-pool pump stations outside the Mikrothives and N. Anchialos. Three soil samples were collected from depths 0.3, 0.7, and 1 m, and radon activity concentration and emanation factor were determined along with similar samples taken in the Thessaloniki radon station.

**Fig. 8:**
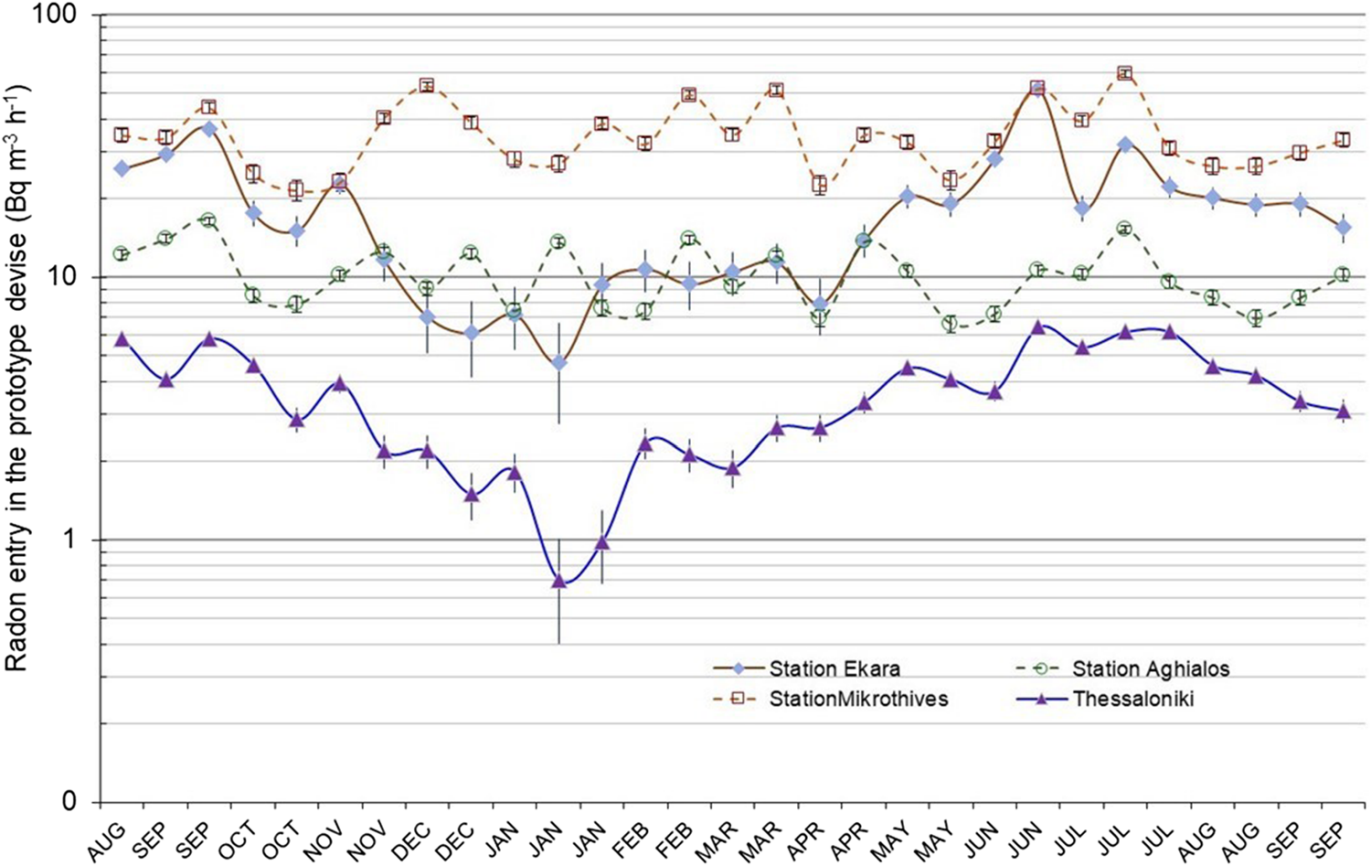
The impact of seismicity on radon entry from the ground in the prototype device

Although radium concentration and radon emanation factor are similar both in Thessaly and Thessaloniki (Table 3), the data demonstrate that the radon entry is quite higher than in Thessaloniki (Fig. 8). The lowest annual average radon entry of 10 Bq m^−3^ h^−1^ appeared in N. Anchialos and the highest 40 Bq m^−3^ h^−1^ in Mikrothives, which remained almost constant due to time. In contrast, the Ekkara station followed a seasonal variation like Thessaloniki’s data ranging between 10 and 50 Bq m^−3^ h^−1^. one order of magnitude higher than, 5 (± 4) Bq m^−3^ h^−1^ such as Thessaloniki. However, a similar variation or higher than 50 Bq m^−3^ h^−1^ has been reported in out-of- areas with underground clay soil (Nero and Nazaroff 1984; UNSCEAR 2000).

**Table 3:** ^226^Ra and ^222^Rn concentration and radon emanation factor in soils in Thessaloniki and along a seismic area

Location	^226^Ra		Emanation factor (%)		Rn emanating	
Ekkara	13	2	23	3	2.9	0.6
Mikrothives	20	3	21	3	4.2	0.9
N. Anchialos	12	2	20	2	2.4	0.5
Thessaloniki	17	3	23	3	4.3	0.9

The radon entry rate was 4–13 times higher, reaching 200 Bq m^−3^ h^−1^, which can increase further in rich uranium-radium underground soil and water, providing constantly high radon entry. However, dwellings cited in areas frequently attacked by earthquakes should have an increased number of cracks and porous openings that increase the total porosity of the dwelling. So, the infiltration rate of the dwelling increases, and the radon level drops indoors.

### Radon entry due to tap water

**Fig. 9:**
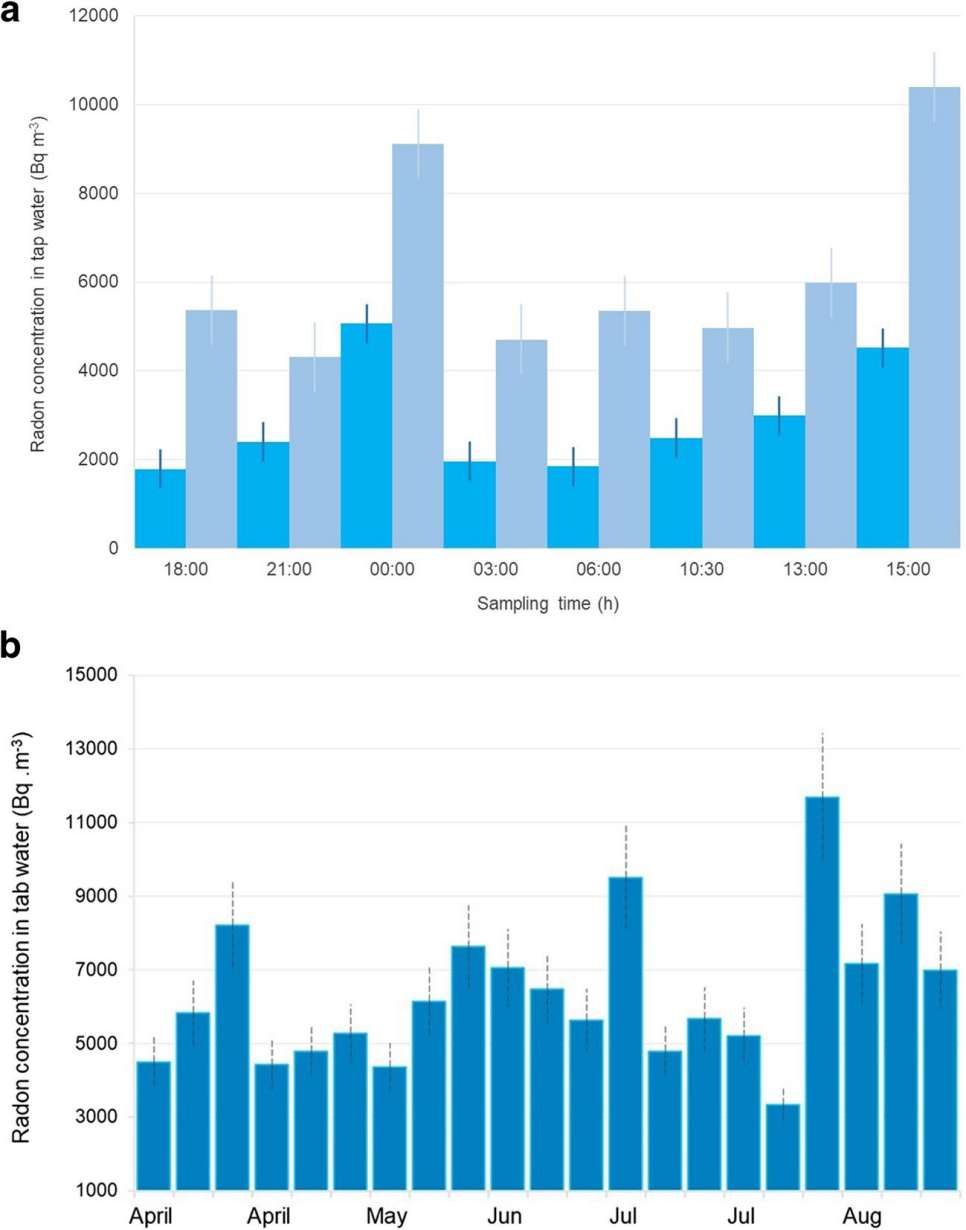
Radon concentration in tap water in Thessaloniki city (**a**) daily variation (dark and light bars represent two different daily experiments) and (**b**) seasonal variation (daily sample at 15:00)

The daily radon variation was studied by collecting eight samples in specific time integrals, as presented in Fig. 9a. The radon level is lower or close to the limit of 11 KB m^−3^, adopted for the public (Nazaroff et al. 1987; UNSCEAR 2000). The radon level reached the maximum at 15:00 and 00:00 when double radon was measured in tap water than the rest of the day when low radon levels were measured. This effect may be attributed to the freshwater entering the public water system supply, giving high radon concentrations. In contrast, the water used is aged with low radon levels for the rest of the day.

The radon concentrations in tap water were measured for 5 months, collecting 21 samples weekly at 15:00 daily. The maximum daily radon concentration levels ranged from 3.3 to 11.7, with a mean value and standard deviation of 6.4 ± 2.0 kBq m^−3^ (Fig. 9b). The radon levels in tap water are among the typical values presented in the literature (UNSCEAR 2000; WHO 2009). The radon entry rate in a dwelling due to tap water usage by the inhabitants can be estimated by considering the assessments presented by Nazaroff et al. (1987). The radon entry rate indoors due to the use of tap water was estimated as 1.3 ± 0.7 Bq m^−3^ h^−1^. These values are lower than the radon entry rate of soil gas up one order of magnitude.

### Radon entry due to soil

A simplified model dwelling was considered to study the indoor radon source potential in a dwelling with a 1-m-depth cellar basement and 2.8 in height living room (Table 2). Based on this model, the radon entry rate due to soil gas has been estimated. In April and October, when no temperature gradient appeared in the soil and the device-dwelling, radon entry due to soil gas occurring only by diffusion is 5 0.0 ± 0.5 Bq m^−3^ h^−1^. Due to the stack effect, the radon entry increases to 9.1 ± 1.9 Bq m^−3^ h^−1^. This radon entry comes only from the cellar basement floor; also, considering the wall with a height of 1 m, a factor of 1.4 should be considered (Robinson and Sextro 1995). Then, the annual indoor radon entry rate due to the soil gas entrance into the basement was estimated at 1.4–12.7 with a mean annual rate of 7.0 ± 0.7 Bq m^−3^ h^−1^. This rate from the ground is not reduced due to the concrete applied at the foundations because the aged concrete has the same diffusion length as the gravel-sand soil (Table 1), while the fresh concrete has the same length as silty sand soil. Therefore, no reduction in soil radon flow indoors is expected due to the application of concrete in the foundations.

### Radon entry due to building materials and outdoor air

The building materials are also an indoor radon source. Estimating the radon entry rate was based on the number and dimensions of the rooms considered and the radon exhalation rate from typical building material. The model dwelling construction (Table 2) and the typical values of the Rn exhalation rate from building materials used in Greece, as reported in a previous study (Stoulos et al. 2003), are presented in Table 4. Based on that publication, the radon entry rate due to building materials was estimated following the construction characteristics of the prototype dwelling as 6.6 ± 1.5 Bq m^−3^ h^−1^. The radon entry rate from the major building materials is among the lowest typical values worldwide (UNSCEAR 2000; 2020/21).

**Table 4:** Mean values (ranges) of measured indoor radon source parameters

Radon source	^226^Ra concentration (Bq kg^−1^)	Exhalation rate (Bq m^−2^ h^−1^)	^222^Rn concentration (Bq m^−3^)	Reference
Soil	17 ± 3 (14–20)	27 ± 22 (5–49)	13,248 ± 6186 (6964–20,276)	This work
Tap water			6457 ± 2017 (3342–11,678)	This work
Outdoor air			5.4 ± 1.7 (4–15)	Stoulos and Ioannidou 2020
Concrete	35 ± 9 ( 8–54)	3.5 ± 2.2 (1.1–6.7)		Stoulos et al. 2003
Clay bricks	35 ± 11 (18–66)	0.2 ± 0.2 (up to 0.4)		Stoulos et al. 2003

The outdoor air has an equilibrium equivalent radon concentration, ranging from 3 up to 9 Bq m^−3^ (Stoulos and Ioannidou 2020), which transforms into radon concentration by dividing with the equilibrium factor (*F*). A typical value of *F* = 0.7 for outdoor air was considered [UNSCEAR 2000]. Then, the radon outdoor levels ranged from 4 to 13 Bq m^−3^ with a mean annual concentration of 5.8 ± 2.7 Bq m^−3^ corresponding to the indoor radon’s natural background like the typical worldwide (UNSCEAR 2000; 2020/21). The radon entry rate was 5.8 ± 2.7 Bq m^−3^ h^−1^ for a mean ventilation ratio of 1 h^−1^.

The estimated radon entry rates are comparable and one order of magnitude higher than the tap water. The values are among the lowest measured worldwide due to low radon potential in the underground soil and the building materials (Nero and Nazaroff 1984; UNSCEAR 2000). The contribution of each radon source to indoor radon level is presented in Table 5**.** The indoor radon activity concentration in the prototype dwelling is estimated at 21 ± 7 Bq m^−3^, considering a mean annual ventilation rate of 1 h^−1^. The mean annual radon concentration in the model dwelling was lower than the geometric mean value of indoor radon levels reported worldwide (UNSCEAR 2000).

**Table 5:** Indoor radon source potential in a model dwelling in a temperate climate

Radon source	Radon entry (Bq m^−3^ h^−1^)	Contribution (%)
Soil	7.0 ± 2.4 (1–14)	34 ± 12
Building materials	6.6 ± 1.5 (3–10)	32 ± 7
Outdoor air	5.8 ± 2.7 (4–13)	28 ± 13
Water supply	1.3 ± 0.7 (0.6–2.1)	6 ± 3

The soil radon entry rate from soil appeared similar to building materials, sparing 1/3 of the total. The natural background due to atmospheric air is slightly lower, at 28%, with the tap water giving the rest 1/3. The results are diverse compared to the contribution of the radon source indoors presented (Font and Baixeras 2003; WHO 2009), where the soil contributes around 40–45%, the building materials and the outdoor air 25–30% each and tap water and gas less than 5%. The low radon content in the soil gas in Thessaloniki city is the main reason for this difference. Once the radon soil gas in the underground soil becomes 35 kBq m^−3^, a typical value for underground soils (UNSCEAR 2000; WHO 2009), the radon entry increases up to 15 kBq m^−3^ h^−1^, and then the soil becomes the main contributor of more than 40%. Then, the building materials and outdoor air give around 25% each, and tap water is the lowest, at 5%.

### Risk assessment of the radon problem

According to the data measured and the estimated radon entry rates, the resident’s risk of lung cancer is < 1–2.5% since the radon level has a mean value of 21 ± 5 Bq m^−3^ (Darby et al. 2005; Krewski et al. 2005; UNSCEAR 2017; 2019) due to them living in a typical one-store model dwelling with a basement founded in a low radon ground soil in Thessaloniki with temperate climate conditions. Even if the inhabitants applied an extremely low ventilation rate, like infiltration rates with the lowest value of 0.1 h^−1^, the radon indoor concentration rises to 200 Bq m^−3^, yet below the international limits of radon indoors (ICRP, 2010, Cinelli et al. 2019), Then, the resident’s risk of lung cancer rises to 24%.

Tap water is the lowest contributor since the upper limit of 11 kBq m^−3^ adopted for public supply (UNSCEAR 2000; WHO 2009) gives levels as reported in the manuscript. The radon entry rate due to soil gas can reach 165 Bq m^−3^ h^−1^ in a seismic area, giving indoor radon levels like the extremely poor ventilation rate of the dwelling. Suppose the dwelling also has an extremely poor ventilation rate; the risk is 10–24%, which is an indoor radon problem. In the case of building materials used in Greece, no radon problem occurs because the radon potential is low. Even if the radon entry increases by one order of magnitude, using materials with extreme radium content and/or emanation factor to construct the total masonry, then, the radon entry is 66 Bq m^−3^ h^−1^, and the resident’s risk of lung cancer is 5–12%. Suppose the worst scenario is an extremely low ventilation rate applied by the residents in the typical dwelling constructed with high-exhalate radon and placed in a seismic area. The indoor radon level could be up to 450 Bq m^−3^, and the risk could be up to 36% due to radon indoors.

European countries have adopted various indoor radon limits over the years, with 200 and 300 Bq m^−3^ being the most recent (Cinelli et al. 2019). In Greece, a limit of 300 Bq m^−3^ is adopted, and the radon problem should occur if, in a normal ventilated dwelling (1 h^−1^), the radon entry rate becomes higher than 300 Bq m^−3^ h^−1^. Then, the risk increases up to 18%. However, the limit of 1.6 mSv per year is adopted by ICRP and UNSCEAR concerning the internal dose due to radon daughters’ inhalation (ICRP, 1994; UNSCEAR 2020/21). The limit gives an equilibrium equivalent radon concentration of 37 Bq m^−3^, transforming into radon concentration by dividing the mean value worldwide of equilibrium factor 0.5 for temperate climates (UNSCEAR 2000). So, the corresponding indoor radon limit becomes 75 Bq m^−3^, and the risk < 5%, both lower than the previous.

## Conclusions

Radon entry in a dwelling depends on indoor radon source potential, climate conditions, construction parameters and residents’ lifestyles. Low radon entry was estimated for a model house in a temperate climate (Mediterranean region). The annual averaged indoor radon entry due to the soil gas appearance into the basement was estimated at 1–13 with a mean rate of 7.0 ± 0.7 Bq m^−3^ h^−1^, while the soil’s radon concentration ranges from 7 to 20 with a mean of 13 ± 6 kBq m^−3^. Therefore, the mean annual infiltration rate for the model dwelling is 0.7 (± 0.5) × 10^−1^ h^−1^ based on the stack effect due to the difference between the indoor and outdoor temperatures. If no soil temperature gradient appeared, the radon entry rate relating only to diffusion is 5.0 ± 0.8 Bq m^−3^ h^−1^, with the advection-drive flow of soil gas varying up ± 4.0 Bq m^−3^ h^−1^. The soil entry rate in the model dwelling from the soil was comparable to that of building materials, accounting for 1/3 of the total radon entry indoors. The natural background due to atmospheric air is slightly lower than soil and building materials, while the tap water is even less, giving both of them the rest 1/3 of the pie. For lifetime indoor exposure to those radon levels, a mortality probability coefficient < 1.2% must be adopted for the inhabitants. The soil radon entry is increased by one order of magnitude if seismic faulting appears in the region, reaching 170 Bq m^−3^ h^−1^. A similar effect occurs if ventilation is limited, rising indoor radon entry levels up to 200 Bq m^−3^ h^−1^. Each exceeding scenario gives an indoor radon concentration below 200 Bq m^−3^, elevating the mortality probability coefficient to 12%. With the overlap of the two scenarios, the radon level could rise above 400 Bq m^−3^, increasing the mortality probability to 24%.

## Figures and Tables

**Table 8:**
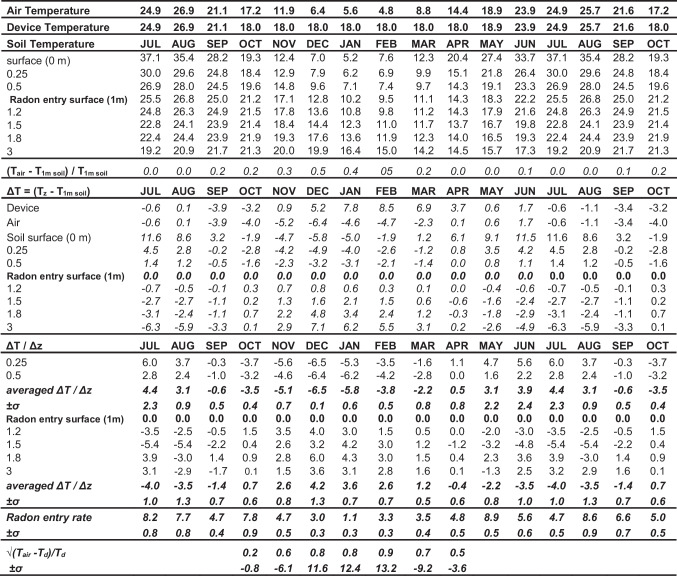
Air and device temperature, the soil temperature gradient to 3 m depth and radon entry rate in a model dwelling in a temperate climate and over a low radon content underground soil

## Data Availability

The author contributes to all issues of this manuscript.

## Annex

Tables contain the raw data of the specific study that was discussed in the manuscript.

Table 6, 7 and 8

**Table 6:** The radon entry rate due to underground soil and various meteorological parameters during the measurements

	Rn entry rate		Temperature		Rainfall	Atm. pressure		Wind speed	
	(Bq m^−3^ h^−1^) ± σ		(°C) ± σ		mm	(+ 760 mmHg) ± σ		(km h^−1^) ± σ	
Jul	5.8	0.6	25.0	3.4	2.1	3.3	1.8	3.4	1.7
Jul	4.1	0.4	25.9	2.2	1.1	5.5	1.9	2.2	1.1
Aug	5.8	0.6	27.3	1.8	0.8	5.5	2.0	1.8	1.0
Aug	4.6	0.6	27.0	1.2	1.3	4.1	2.2	1.2	0.6
Sep	2.9	0.3	22.4	3.2	2.5	6.8	3.2	3.2	2.0
Sep	3.9	0.4	20.0	1.6	2.2	8.8	2.2	1.6	0.8
Oct	5.6	0.7	18.7	3.4	2.5	4.5	2.7	3.4	2.0
Oct	5.2	0.6	15.6	1.6	1.6	2.6	3.5	1.6	0.8
Nov	4.8	0.6	15.1	1.8	2.7	7.5	3.3	1.8	1.0
Nov	2.2	0.3	10.7	4.2	2.4	3.9	5.3	4.2	2.1
Dec	2.2	0.2	8.6	3.8	2.7	4.9	6.0	3.8	1.9
Dec	1.5	0.2	7.6	3.2	2.6	8.0	4.7	3.2	1.6
Jan	1.8	0.2	2.5	4.0	1.5	10.4	5.0	4.0	2.0
Jan	0.7	0.1	6.4	3.2	2.4	7.2	2.4	3.2	1.6
Jan	1.0	0.1	6.2	4.6	2.6	5.3	5.9	4.6	2.3
Feb	2.3	0.2	5.8	4.6	2.5	9.8	4.1	4.6	2.3
Feb	2.1	0.2	4.6	4.4	2.3	2.9	5.6	4.4	2.2
Mar	1.9	0.2	6.0	5.2	3.3	1.4	7.4	5.2	2.6
Mar	2.7	0.3	12.1	4.4	2.2	0.1	5.4	4.4	2.2
Apr	3.9	0.4	13.0	3.8	2.4	3.0	2.7	3.8	1.9
Apr	2.7	0.3	15.2	3.8	1.4	0.8	2.9	3.8	1.9
May	6.3	0.7	16.4	3.2	1.1	2.1	1.6	3.2	1.6
May	5.6	0.7	19.5	3.6	2.2	3.3	3,0	3.6	1.8
Jun	3.3	0.4	23.3	3.8	1.6	2.3	1.7	3.8	1.9
Jun	4.5	0.5	24.1	4.0	2.2	1.4	2.6	4.0	2.0
Jul	4.1	0.5	25.3	5.2	2.1	2.0	2.4	5.2	2.6
Jul	3.7	0.5	24.3	4.8	2.0	2.1	2.7	4.8	2.4
Jul	6.4	0.9	25.8	4.2	1.2	1.3	2.2	4.2	2.1
Aug	5.4	0.6	25.5	4.2	0.9	1.4	2.2	4.2	2.1
Aug	6.2	0.8	25.1	3.8	2.6	4.1	5.7	3.8	1.9
Sep	4.6	0.5	22.1	3.6	2.5	2.5	3.3	3.6	1.8
Sep	4.2	0.4	21.3	3.0	2.3	0.7	3.9	3.0	1.5
Oct	3.4	0.3	19.3	3.4	1.5	0.3	2.5	3.4	1.7
Oct	3.1	0.3	19.7	3.0	1.6	2.8	2.7	3.0	1.5

^6^,

**Table 7:** The radon entry rate and radon concentration of the underground soil associated with the infiltration rate in the prototype device

	Radon in soil gas (kB m^−3^)		Radon entry rate (B m^−3^ h^−1^)		Infiltration rate (h^−1^)	
Jul	11.2	1.7	5.8	0.9	0.069	0.015
Jul	6.9	1.2	4.1	0.7	0.079	0.018
Aug	10.3	1.7	5.8	0.9	0.076	0.017
Aug	9.3	1.6	4.6	0.8	0.067	0.016
Sep	9.1	1.3	2.9	0.4	0.042	0.008
Sep	7.7	1.3	3.9	0.7	0.068	0.016
Oct	10.9	1.6	5.6	0.8	0.069	0.014
Oct	9.6	1.5	5.2	0.8	0.073	0.016
Nov	8.7	1.2	4.8	0.7	0.074	0.014
Nov	9.4	1.2	2.2	0.3	0.031	0.005
Dec	14.4	1.9	2.2	0.3	0.020	0.004
Dec	8.8	1.2	1.5	0.2	0.023	0.004
Jan	18.7	1.7	1.8	0.2	0.013	0.002
Jan	13.2	1.7	0.7	0.1	0.007	0.001
Jan	15.3	1.7	1.0	0.1	0.009	0.001
Feb	17.2	1.7	2.3	0.2	0.018	0.003
Feb	19.4	2.3	2.1	0.3	0.015	0.002
Mar	13.9	1.1	1.9	0.2	0.018	0.002
Mar	16.4	1.7	2.7	0.3	0.022	0.003
Apr	20.2	2.3	3.9	0.4	0.026	0.004
Apr	15.1	1.7	2.7	0.3	0.024	0.004
May	16.4	1.7	6.3	0.7	0.051	0.008
May	15.9	1.7	5.6	0.6	0.047	0.007
Jun	13.1	1.1	3.3	0.3	0.034	0.004
Jun	11.0	1.1	4.5	0.5	0.054	0.008
Jul	12.2	1.1	4.1	0.4	0.044	0.006
Jul	12.9	1.8	3.7	0.5	0.038	0.008
Jul	12.4	1.8	6.4	0.9	0.069	0.014
Aug	11.5	1.3	5.4	0.6	0.063	0.010
Aug	13.5	1.3	6.2	0.6	0.061	0.008
Sep	14.2	1.9	4.6	0.6	0.043	0.008
Sep	10.2	1.8	4.2	0.7	0.055	0.014
Oct	9.2	1.2	3.4	0.4	0.048	0.009
Oct	11.0	1.8	3.1	0.5	0.037	0.009

^7^ and 8
